# Aberrant cortico-thalamo-cerebellar network interactions and their association with impaired cognitive functioning in patients with schizophrenia

**DOI:** 10.1038/s41537-023-00375-8

**Published:** 2023-08-12

**Authors:** Minji Ha, Soo Hwan Park, Inkyung Park, Taekwan Kim, Jungha Lee, Minah Kim, Jun Soo Kwon

**Affiliations:** 1 Department of Brain and Cognitive Sciences, Seoul National University College of Natural Sciences, Seoul, Republic of Korea; 2 Geisel School of Medicine at Dartmouth, Hanover, NH USA; 3 Department of Bio and Brain Engineering, Korea Advanced Institute of Science and Technology, Daejeon, Republic of Korea; 4 Department of Neuropsychiatry, Seoul National University Hospital, Seoul, Republic of Korea; 5 Department of Psychiatry, Seoul National University College of Medicine, Seoul, Republic of Korea; 6 Institute of Human Behavioral Medicine, SNU-MRC, Seoul, Republic of Korea

**Keywords:** Biomarkers, Neural circuits

## Abstract

Evidence indicating abnormal functional connectivity (FC) among the cortex, thalamus, and cerebellum in schizophrenia patients has increased. However, the role of the thalamus and cerebellum when integrated into intrinsic networks and how those integrated networks interact in schizophrenia patients are largely unknown. We generated an integrative network map by merging thalamic and cerebellar network maps, which were parcellated using a winner-take-all approach, onto a cortical network map. Using cognitive networks, the default mode network (DMN), the dorsal attention network (DAN), the salience network (SAL), and the central executive network (CEN) as regions of interest, the FC of 48 schizophrenia patients was compared with that of 57 healthy controls (HCs). The association between abnormal FC and cognitive impairment was also investigated in patients. FC was lower between the SAL-CEN, SAL-DMN, and DMN-CEN and within-CEN in schizophrenia patients than in HCs. Hypoconnectivity between the DMN-CEN was correlated with impaired cognition in schizophrenia patients. Our findings broadly suggest the plausible role of the thalamus and cerebellum in integrative intrinsic networks in patients, which may contribute to the disrupted triple network and cognitive dysmetria in schizophrenia.

## Introduction

Schizophrenia is an illness characterized by deteriorating functioning, including positive symptoms, negative symptoms, affective symptoms, and cognitive symptoms^1^. Distinctive from other symptoms, cognitive deficits emerge in the early phases of the disorder and even before the onset of the illness^2–4^. Cognitive symptoms manifest across various domains of cognition ranging from processing speed, working memory, executive functioning, and attention^5–7^, drastically altering the quality of life of patients^8,9^. As a means to comprehend the underlying mechanism of the functional decline in schizophrenia patients, Andreasen et al.^10^ proposed a theoretical model, “cognitive dysmetria,” hypothesizing that abnormal coordination among the prefrontal cortex, thalamus, and cerebellum may contribute to the functional deficits shown in patients.

However, during the past two decades of advancement in neuroimaging techniques, the thalamus and the cerebellum have been generally overlooked due to the bias that prioritizes the cerebrum and the long-standing impression of their role as a sensory and movement-related relay ancillary to the cerebral cortex^11,12^. The importance of the thalamus and cerebellum in interacting with cortical regions is demonstrated in a neuroanatomical model supporting the hypothesis that the cerebellum, thalamus and cortical areas create a closed loop that receive and project inputs when individuals process complex functions. In recent years, a substantial body of literature has suggested that the thalamus^13–16^ and cerebellum^17–19^ are engaged in information modulation interacting with other cortical areas not only limited to basic sensory processing but also including complex cognitive processing. Lesion studies have shown that damage to the thalamus and cerebellum may lead to certain cognitive impairments, and recent animal studies have found cerebellar influence on cortical coherence via communication via its thalamic pathway^20–25^. In addition, cortico-thalamic and cortico-cerebellar interactions in healthy populations have been known to be associated with specific cognitive functions^26,27^.

In patients with schizophrenia, atypical interconnectivity among the cortex, thalamus, and cerebellum can be distinctly inferred from both structural studies using diffusion tensor imaging^28–31^ and functional studies. While functional dysconnectivity between the cortex and the entire thalamus as a region of interest (ROI) has been relatively well established^32–34^, studies have used segmented thalamic ROIs based on the functional connectivity (FC) of each thalamic voxel to anatomically parsed cortical lobes. These studies have revealed consistent dysconnectivity with posterior cerebellar clusters and the cortical areas that are often referred to as the nodes of higher-order cognitive networks in not only chronic schizophrenia patients^35,36^ but also first-episode psychosis (FEP) and clinical high-risk (CHR) patients^37–39^.

Nevertheless, these earlier studies have only investigated the FC among the cortex, thalamus, and cerebellum at the cluster level, overlooking the importance of understanding the underlying pathophysiology at the network level. Higher-order cognitive networks, including the default mode network (DMN), salience network (SAL), central executive network (CEN), and dorsal attention network (DAN), are suggested to be engaged in a wider range of complex cognition^40–44^. However, patients with schizophrenia have shown abnormal functional interactions within and among these cortical intrinsic networks, often correlated with poor cognitive functioning^45–51^, indicating abnormal orchestration of the brain on a large scale. Notably, efforts have been made to apply the functional network properties of the cerebellum and thalamus^52–55^ and have found dysfunctional thalamic and cerebellar connectivity patterns to cortical networks in patients with schizophrenia^56,57^.

However, despite ample evidence suggesting crucial interactions between the thalamus and cerebellum with cortical intrinsic networks, the role of the thalamus and cerebellum when both are considered composed of higher-order cognitive networks and their association with cognitive dysfunction as integrative networks in schizophrenia remain unclear. Here, we aimed to probe the functional network interactions in patients with schizophrenia by incorporating thalamic and cerebellar network maps that had been created based on their functional relevance to the cortical networks into intrinsic networks and creating integrative cortico-thalamo-cerebellar (CTC) networks. We hypothesized that the functional interactions within the CTC networks would be significantly lower in patients with schizophrenia than in healthy controls (HCs). We further performed an exploratory investigation into whether altered network interactions are associated with impaired cognitive performance in patients.

## Results

### Participant characteristics

The demographic, clinical, and cognitive characteristics of the participants are shown in Table 1. There was no significant difference in sex (χ^2^ = 0.02, *p* = 0.902) between schizophrenia patients and HCs, while there was a significant group difference in age (t = −5.17, *p* < 0.001) and intelligence quotient (IQ) (t = 3.51, *p* < 0.001). With age as a covariate, schizophrenia patients demonstrated significantly poorer performance on the Stroop Color Word Test – color word inference (SCW – CW) measure (F = 13.45, *p* < 0.001), indicating that schizophrenia patients had more difficulty suppressing the irrelevant word meaning and focusing on the ink color than the controls, resulting in less accurate responses.

**Table 1:** Demographic, clinical, and cognitive characteristics of all subjects.

Variables	Schizophrenia patients (*n* = 48)	HCs (*n* = 57)	Statistics	
			t/χ^2^/F	p
Demographic				
Age (years)	34.21 ± 8.20	25.75 ± 8.47	−5.17	<0.001^**^
Sex (male/female)	25/23	29/28	0.02	0.902
IQ	100.92 ± 16.10	112.95 ± 9.79	3.51	<0.001^**^
Duration of illness (months)	158.96 ± 75.47	–	–	–
Clinical				
PANSS total	69.89 ± 20.20	–		
Positive	18.96 ± 5.73	–		
Negative	17.48 ± 5.97	–		
General	33.46 ± 10.75	–		
HAM-A	39.87 ± 15.46	–		
HAM-D	6.22 ± 5.33	–		
BPRS	10.04 ± 6.42	–		
Neurocognitive				
SCW-CW	−0.55 ± 0.84	0.38 ± 0.93	13.45	<0.001^**^

Note. Data are presented as the mean ± standard deviation. Independent t tests were used for continuous variables, and Welch’s t test was used if the variances were not equal. Chi-square tests for categorical data were performed. The neurocognitive test scores were converted into Z-standardized scores and were compared with one-way analysis of covariance with age as the covariate.

*BPRS* Brief psychiatric rating scale, *CW* Color word interference, *HAM-A* Hamilton rating scale for anxiety, *HAM-D* Hamilton rating scale for depression, *HCs* healthy controls, *IQ* Intelligence quotient, *PANSS* Positive and Negative Syndrome Scale, *SCW* Stroop color word test.

*p < 0.01.

**p < 0.001.

### Network assignment

The cortical network map was generally similar to the previous study^58^, which was created using one thousand subjects. The prefrontal and parietal areas were mostly shared across higher-order cognitive networks. The cortical regions of the DMN include dorsolateral prefrontal, precuneus, posterior cingulate, orbitofrontal, and parahippocampal areas, with DMN 3 representing an area more distinctively in the right angular gyrus. The DAN was represented in the supramarginal gyrus, superior parietal and frontal, and middle temporal gyrus. The SAL was represented in the insula, anterior cingulate (ACC), and central opercular regions; SAL 1 was shown in the supplementary motor cortex as well, and SAL 2 was represented in dorsolateral and ventrolateral prefrontal areas with larger clusters in the dorsal ACC. The four CEN clusters were characterized primarily in the posterior cingulate areas, dorsal precuneus, middle frontal, lateral orbitofrontal, and temporoparietal regions.

**Fig. 1:**
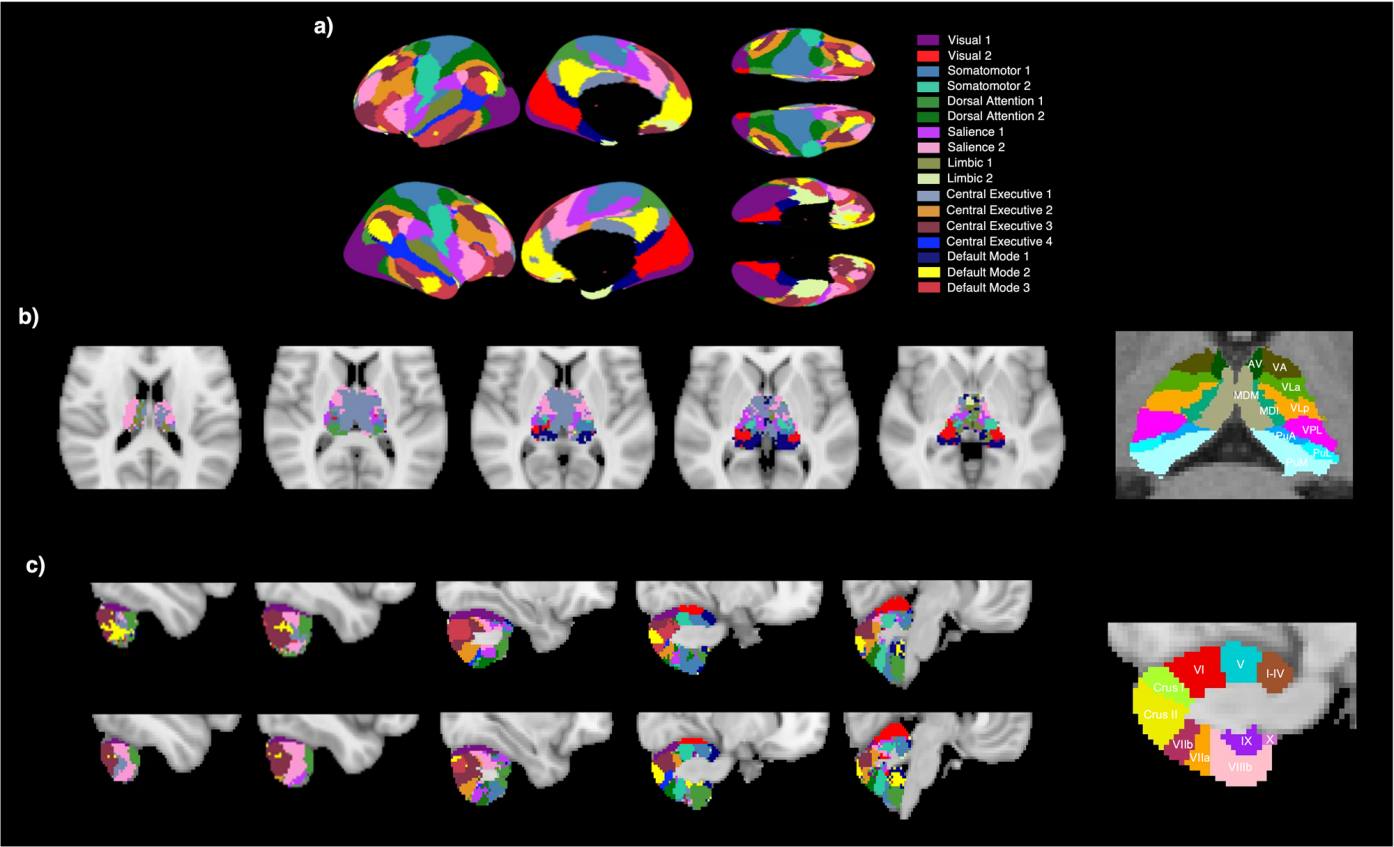
Cortical network parcellation map of 17 networks, a thalamic network parcellation map, and a cerebellar network parcellation map. **a** Cortical network parcellation using the methods from a previous study^58^. **b** Thalamic functional network parcellation with anatomical parcellations of thalamus^59^. **c** Cerebellar functional network parcellation with anatomical parcellations of cerebellum^60^. AV anteroventral, VA ventral anterior, VLa ventral lateral anterior, VLp ventral lateral posterior, VPL ventral posterior, MDl mediodorsal lateral parvocellular, MDM mediodorsal medial magnocellular, PuA pulvinar anterior, PuL pulvinar lateral, PuM pulvinar medial.

As shown in Fig. 1c, the four higher-order cognitive networks showed associations mostly with the posterior region of the bilateral cerebellum. Lobule VIIb was bilaterally reflected in the DAN, while a large sector of the SAL was demonstrated in lobules VI and VIIA, including Crus I and Crus II. Smaller clusters of the CEN were represented across Crus I, Crus II, and VIIB, but CEN 3 shared larger areas of Crus II. Lobule X was represented by DMN 1, and lobule IX was represented by DMN 2. DMN 2 and 3 were mostly reflected in Crus I and II.

Considering the smaller size of the cortex and cerebellum, the thalamus was shared less across networks. The SAL was reflected in mediodorsal (MD) and ventral nuclei, including the ventral anterior (VA), ventral lateral (VL), and ventral posterior (VP) regions. DAN 1 was represented in the pulvinar. A large MD cluster was characterized by CEN 1, and small clusters of the DMN were in the posterior areas of the thalamus, including the pulvinar. DAN 2, CEN 2–4, and DMN 2-3 were not represented in the thalamus. The final network map of the cortex, thalamus, and cerebellum is shown in Fig. 1, and anatomical parcellations of thalamus^59^ and cerebellum^60^ are also presented for reference.

### Between-network ROI-to-ROI connectivity

**Fig. 2:**
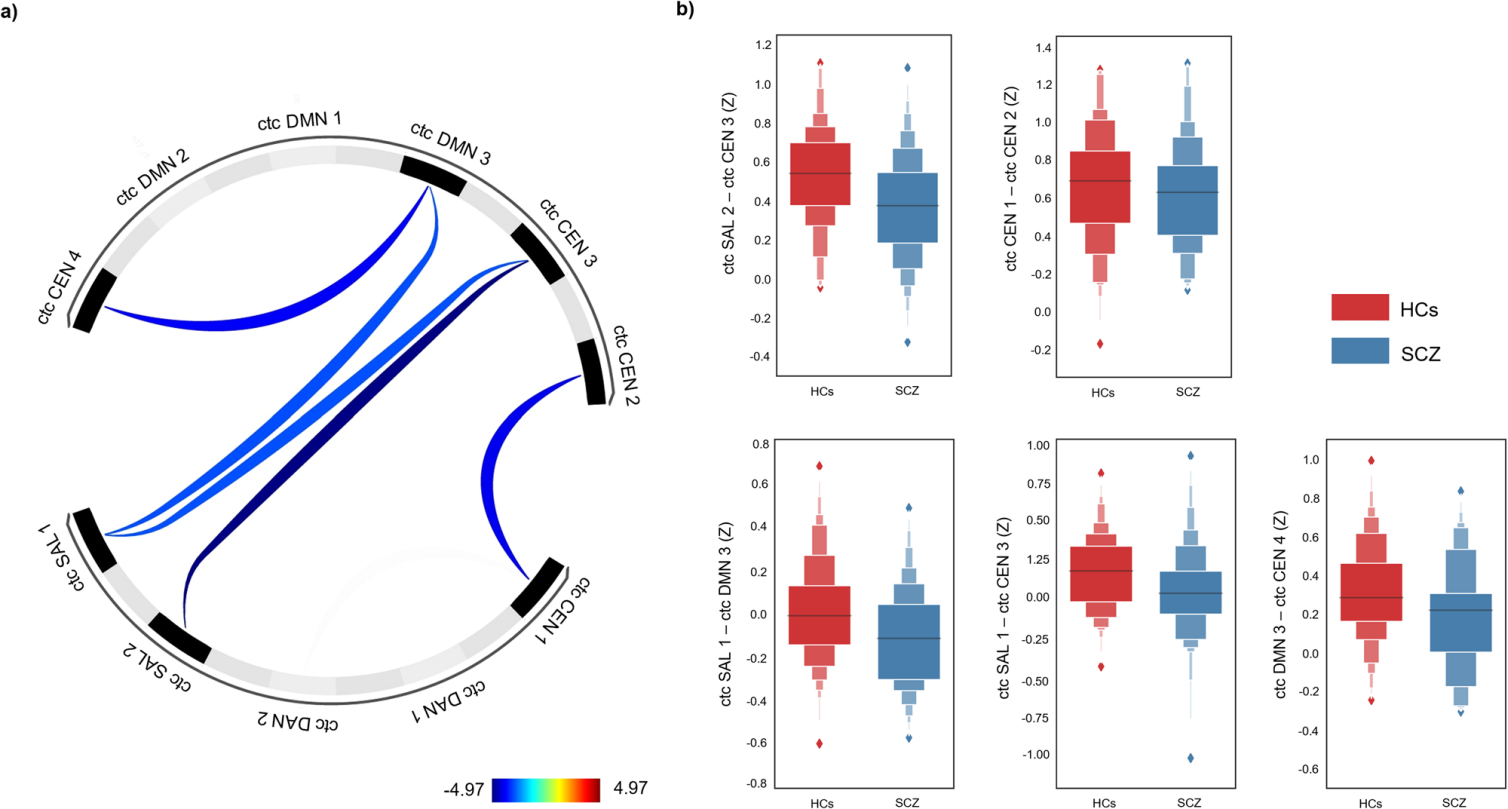
Abnormal cortico-thalamo-cerebellar network ROI-to-ROI interactions in schizophrenia patients compared to those in HCs. Between-group differences with age controlled as a covariate thresholded at cluster p-FDR level < 0.05 and connection p-FDR level < 0.05 in (**a**) a ROI-to-ROI connectivity matrix with the t scores displayed on the color bar and (**b**) boxplots with the y-axis representing the z scores from FC between network ROIs. ctc cortico-thalamo-cerebellar, DMN default mode network, CEN central executive network, SAL salience network, DAN dorsal attention network, HCs healthy controls, SCZ schizophrenia patients.

Among 55 connections between all pairs of ROIs, 5 pairs of ROIs that were included in two clusters exhibited statistically significant group differences. The schizophrenia group showed decreased connectivity compared to the HC group. Connectivity between SAL 2 and CEN 3 was lower in patients (cluster level *p*-FDR < 0.001; connection level *p*-FDR < 0.001), and the connectivity between CEN 1 and CEN 2 was also lower in patients (cluster level *p*-FDR < 0.001; connection level *p*-FDR < 0.001). In the schizophrenia group, SAL 1 showed lower FC with DMN 3 (cluster level *p*-FDR < 0.001; connection level *p*-FDR = 0.003) and with CEN 3 (cluster level *p*-FDR < 0.001; connection level *p*-FDR = 0.003). Finally, the FC between DMN 3 and CEN 4 was decreased in the schizophrenia group compared to that the HC group (cluster level *p*-FDR = 0.003; connection level *p*-FDR = 0.002). DAN did not show a significant difference from any other networks. The mean network FC values and the connectivity strengths between patients and controls are shown in Table 2 and Fig. 2.

**Table 2:** Cortico-thalamo-cerebellar between-network connectivity significantly differed between schizophrenia patients and HCs when controlling age as a covariate.

Functional network connectivity	Schizophrenia patients	HCs	FC strengths	
			Cluster p-FDR corrected	Connectivity p-FDR corrected
Cluster A			<0.001^**^	
SAL 2 – CEN 3	0.367 ± 0.282	0.521 ± 0.251		<0.001^**^
CEN 1 – CEN 2	0.608 ± 0.280	0.662 ± 0.312		<0.001^**^
SAL 1 – DMN 3	−0.122 ± 0.229	0.011 ± 0.236		0.003^*^
SAL 1 – CEN 3	0.031 ± 0.296	0.156 ± 0.237		0.003^*^
Cluster B			0.003^*^	
DMN 3 – CEN 4	0.181 ± 0.275	0.308 ± 0.248		0.002^*^

Note. Data are presented as the mean ± standard deviation.

*FC* functional connectivity, *HCs* healthy controls, *FDR* false discovery rate, *DMN* default mode network, *CEN* central executive network, *SAL* salience network.

*p < 0.05.

**p < 0.001.

### Correlation between network interactions and neurocognitive performance in patients

**Fig. 3:**
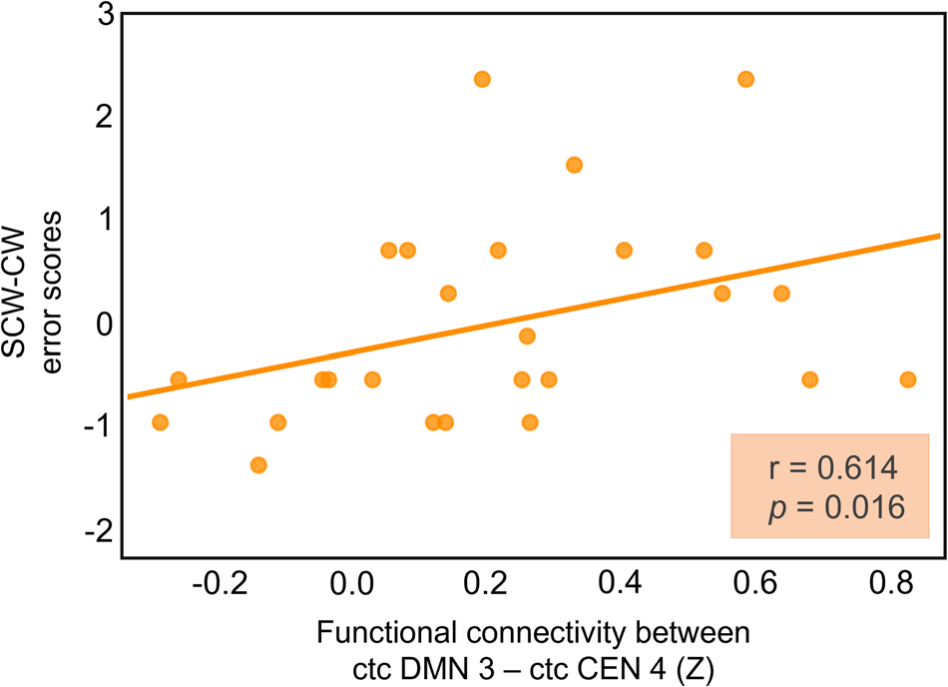
Partial correlations between cortico-thalamo-cerebellar network ROI-to-ROI connectivity and neurocognitive performance in patients with schizophrenia. Regression plot of partial correlations controlling for age as a covariate. The x-axis represents the z scores from between-network ROI connectivity, and the y-axis represents the correlated neurocognitive test performance. SCW Stroop Color and Word Test, CW color word inference, ctc cortico-thalamo-cerebellar, DMN default mode network, CEN central executive network.

Given the significant differences observed between schizophrenia patients and HCs in the 5 pairs of functional connectivity of CTC network ROIs, we further investigated the relationship between impaired performance on CW trials and disrupted connectivity in schizophrenia. Controlling for IQ, the DMN 3 and CEN 4 interaction demonstrated a statistically significant correlation with the SCW-CW performance of schizophrenia patients. No other network interactions were correlated with the SCW-CW performance. When FDR-corrected for multiple comparisons, the correlation between hypoconnectivity between DMN 3 and CEN 4 and SCW-CW performance (*r* = 0.614, *p* = 0.016) remained significant (Fig. 3).

## Discussion

The present study aimed to understand the pathophysiology of schizophrenia by integrating the thalamus and cerebellum with cortical intrinsic networks and exploring the functional differences between CTC networks in schizophrenia patients. We observed significantly lowered FC between the SAL and CEN, between the SAL and DMN, between the DMN and CEN, and within the CEN in schizophrenia patients. In addition, the CTC network interactions between the DMN and CEN were correlated with impaired cognition in schizophrenia patients. Our findings demonstrated plausible roles of the thalamus and cerebellum in cortical network interactions and suggest abnormal interactions among the cortex, thalamus, and cerebellum at the network level.

We generated thalamic and cerebellar network maps to represent our own functional data. The cerebellar parcellation result was in line with previous studies that delineated the cerebellum based on its FC to the cerebral cortex^54,61–63^, as it shows clear and distinctive representations of the DMN, CEN, SAL, and DAN in Crus I, Crus II, lobule VIIB, and lobule IX. Extensive evidence from anatomical and functional imaging studies has consistently demonstrated that the posterior lobe, including lobules VI and VII, is involved in nonmotor processing, whereas the anterior lobe is associated with sensorimotor processing^17,18,64^. The MD thalamic nucleus was mostly represented by the CEN, sharing some clusters with the SAL. This seems to be a reasonable representation considering the major anatomical projections to the prefrontal cortex and orbitofrontal cortex and accounting for the functional associations with various cognitive functions, including working memory and executive function^13,14,16,20,65^. In addition, the DAN was only reflected in the pulvinar region. This finding generally correlates with the thalamic pattern associated with cortical resting-state networks^26,53,66^.

We found reduced connectivity across the CTC-SAL, CTC-CEN, and CTC-DMN in patients with schizophrenia. Foremost, this result agrees with previous findings regarding network interactions at the cortical level, and dysconnectivity, generally hypoconnectivity within these cortical networks, has been consistently reported in the literature^47,49,67^. Looking at the thalamic FC to the cortex and cerebellum, respectively, in schizophrenia patients, decreased thalamic connectivity between the DMN cerebellar cluster and CEN cerebellar cluster was reported, and its connectivity to both the cortical and cerebellar DMN and CEN became unstable after the onset of the illness^68^. Additionally, although Kim et al.^56^ found both hypo- and hyperconnectivity between thalamic network clusters and the cortical network in FEP individuals, CHR individuals, and unaffected relatives of schizophrenia patients, thalamic dysconnectivity at the network level and larger engagement of the thalamus in cortical network dysfunction were clearly suggested. Furthermore, when measuring the connectivity between the 17 cortical networks and the cerebellum^45^, decreased connectivity between higher-order cognitive networks, including the SAL, DMN, and CEN, and posterior cerebellar areas, including Crus I/II, VI, IX, and Vermis VIIa, were reported in schizophrenia patients. Given that our results align with those of previous studies investigating thalamic and cerebellar connectivity to cortical resting networks, disrupted involvement of the thalamus and cerebellum at the brain network level on a large scale can be suggested in schizophrenia patients.

In addition, we found a correlation between performance in the inference condition of the Stroop Color Word Test and network connectivity between the CTC-DMN and CTC-CEN. Cognitive deficits, especially working memory deficits, in schizophrenia patients have been associated with the cerebellar and thalamic regions of the DMN and CEN, as shown in our current study, as well as the connectivity between the cortical level of the DMN and CEN^49,50^. Posterior areas of the cerebellum and higher-level thalamic nuclei have been repeatedly suggested to be involved in complex human cognition, and regions such as the temporal gyrus, frontal pole, and cerebellar Crus I and II often coactivate and demonstrate aberrant FC with the whole thalamus while schizophrenia patients perform working memory tasks^69–71^. Accordingly, the correlation observed in our analysis between CTC-DMN and CTC-CEN hypoconnectivity and the impaired SCW-CW performance in patients are in line with the literature, suggesting their influence on flawed cognitive processing in patients.

Moreover, it is crucial to note that the hypoconnectivity among the CTC-SAL, CTC-CEN, and CTC-DMN supports the triple network model in psychopathology. The triple network model suggests that in psychosis, SAL failure to detect salient stimuli and its inappropriate mediation and modulation of the engagement between a task-negative network, the DMN, and a task-positive network, the CEN, eventually lead to impaired functioning in the patients^72,73^. Such abnormal cortical network interactions, often hypoconnectivity, have been repeatedly reported in schizophrenia patients^72,74^, FEP patients^51,75^, and CHR individuals^51,76^ as well. It is noteworthy to mention that in triple network dynamics, the SAL, also known as the ventral attention or cingulo-opercular network, plays a crucial role in detecting and interpreting salient stimuli and modulating two other networks depending on its interpretation. The crux of the matter is that the malfunctioning of the SAL and the inaccurate salience attribution in schizophrenia patients indicate misinterpretation and ineffective filtering of the significance of the stimuli, which are related to the wide range of symptoms, including cognitive impairments in patients. A meta-analysis on 56 resting FC datasets in schizophrenia patients revealed network dysconnectivity within the CEN, DMN, thalamus network, and emotion network that includes the cerebellum; moreover, the SAL seemed to be in the center of these networks, demonstrating disarray with all other networks^49^. Our study took the characteristics of the thalamus and cerebellum with the cortical network into account, resulting in hypoconnectivity among these three networks aligned with the current literature on large-scale network dysconnectivity in schizophrenia patients.

Furthermore, in light of the recent spotlight on the modulatory function of the thalamus^14^ and cerebellum^25^ over cortical areas, particularly cortical coherence, further investigation into the possible involvement of the thalamus and cerebellum in SAL dysfunction may provide valuable insights. Hwang et al. ^53^ revealed both provincial and connector hub properties of the thalamus, indicating the dynamic and active role of the thalamus in communicating and modulating the interactions between and within the functional networks. In addition, as the neural circuit matures, the cerebellum develops as a solid hub and establishes functional links with various cortical intrinsic networks over time, communicating on a large scale and contributing to cognitive processing as a hub entity^77,78^. Within this context, although we can only speculate based on the limited literature, the triple network dysconnectivity in psychosis may extend to the thalamus and cerebellum, possibly affecting the modulatory function among large-scale networks.

Our study had a few limitations to consider. First, our study samples were not matched in age. We tried to control the effect of age between the two groups as a covariate, but this aspect of the study should be noted when interpreting the results. Second, we investigated the network connectivity differences using the CTC network map as a whole ROI mask. Although our approach was able to provide a crude picture of how CTC interactions are disrupted in patients at the network level, information regarding the causality of the network interactions cannot be inferred from our findings. Further work exploring the detailed mechanism of thalamic and cerebellar modulation in large-scale networks seems to be essential in future work. Third, although the present study secured 246 resting fMRI data volumes, the total scan time was closer to the minimum required time, which is 5.2 minutes^79^. As this scan time may increase the potential risk of obscuring the signals, the present results should be interpreted with care. Finally, our study primarily focused on investigating network-level CTC interactions, hence the preliminary correlation with SCW-CW performance. Investigation of the CTC network interactions with a wide range of cognitive functioning in schizophrenia patients is encouraged in the future.

Schizophrenia is a large-scale brain disorder that is not caused solely by regional impairment. The findings of the present study demonstrated functional dysconnectivity at the network level instead of the cluster level and further suggested the possibly poor contribution of the thalamus and cerebellum, regions that have been underappreciated due to the corticocentric myopia. Therefore, our results support two important models of psychopathology, cognitive dysmetria and the triple network model; furthermore, our findings could provide an additional foundation for future studies to incorporate the role of the thalamus and cerebellum in network interactions into the investigation of the pathophysiology of schizophrenia.

## Methods

### Participants

A total of 105 subjects were included in the present study, including 48 schizophrenia patients and 57 HCs. The patients were recruited from the outpatient clinic of the Seoul National University Hospital (SNUH), Korea. The diagnosis of schizophrenia and schizoaffective disorder was made according to the Structured Clinical Interview for Diagnostic and Statistical Manual of Mental Disorders, Fourth Edition Axis I Disorders (SCID-I). HCs were recruited from SNUH via internet advertisements. The exclusion criteria for the control group were a total IQ score below 70, history of severe head trauma and substance abuse not including nicotine, medical illness that might have entailed psychotic symptoms and cognitive impairments and having 1^st^ – 3^rd^ relatives who had a history of psychotic disorder. HC participants were also screened for any psychiatric symptoms via the Structured Clinical Interview for DSM-IV-Non-Patient Version (SCID-NP). The exclusion criteria for patients included substance abuse or dependence (not including nicotine), neurological diseases or significant head trauma, medical illness that might have entailed psychiatric symptoms, and intellectual disability. The participants’ IQ levels were assessed using the Korean version of the Wechsler Adult Intelligence Scale^80^. Specifically, the Doppelt short form^81^ was used, which utilizes four subtests, including arithmetic, vocabulary, picture arrangement, and block design. The IQ test was performed outside the scanner. The current data from the participants have not been used in any previous studies. All assessments were made by trained and certified clinicians, and all participants were given a written informed consent form before participating in the study (IRB no. H-1110-009-380). The study was conducted in accordance with the Declaration of Helsinki and was approved by the IRB of the SNUH (IRB no. H-2212-048-1384).

### Image acquisition and preprocessing

All participants were scanned using a 3 T Trio MR scanner (Siemens Magnetom Trio, Erlangen, Germany) using a 32-channel head coil. The resting fMRI data were acquired with a gradient echoplanar imaging (EPI) pulse sequence with the following parameters: repetition time/echo time (TR/TE) = 1500/30 ms, flip angle = 85°, field of view (FOV) = 256 mm, and voxel size = 2.3 × 2.3 × 2.3 mm^3^. For spatial distortion correction, field maps were scanned with EPI with the following parameters: TR/TE = 4120/30 ms, flip angle = 85°, FOV = 256 mm, and voxel size = 2.3 × 2.3 × 2.3 mm^3^ in opposing phase encoding directions (right > left and left > right). During the acquisition of resting-state fMRI data, participants were asked to keep their eyes closed but not to fall asleep. They were also instructed to avoid making any movements during the acquisition, and head cushions were used to secure the participants’ positions and minimize their motions. The total acquisition time for blood oxygen level-dependent (BOLD) fMRI images was 6 minutes and 44 seconds. T1 images were obtained for anatomical reference with magnetization-prepared rapid gradient echo (MP-RAGE) with the following parameters: TR/TE = 2400/2.19 ms, flip angle = 8°, FOV = 272 mm, and voxel size = 0.8 × 0.8 × 0.8 mm^3^. All the images were manually inspected for any visible motions, possible artifacts, and intact cerebella.

We preprocessed the functional data using the fMRI preprocessing pipeline, which was implemented with FreeSurfer, FSL, and customized MATLAB functions. The first four volumes were removed, yielding a total of 246 volumes, and slice timing was corrected in interleaved order via fsl_slicetimer. Motion correction was performed mainly via the fsl_motion_outliers function, calculating the framewise displacement (FDRMS) with thresholding at <0.5. Spatial distortion was corrected using field map brain masks from the opposite phase encoding direction. Using FSL bbregister, each BOLD image was registered to the T1 structural data for the subject. After obtaining white matter, cerebrospinal fluid, and whole-brain masks, nuisance signals from these and the motion parameters were regressed out and removed. Temporal bandpass filtering was applied at 0.008 < f < 0.09 Hz. For cortical parcellation, we continued with spatial normalizations from native space to fsaverage5.

### Cortical network parcellation

In preparation for cortical network parcellation, the preprocessed data in native space were spatially normalized to fsaverage5 surface space and spatially smoothed to a 6 mm full-width half-maximum (FWHM) isotropic Gaussian kernel. Cortical parcellation was undertaken as demonstrated in a previous study^58^. As we describe the process briefly here, more details can be found in Yeo et al. ^58^ Each hemisphere with normalized cortical data that were projected to fsaverage5 space had 10,242 vertices and 1,175 ROIs across the space mesh. The connectivity profile of each subject was calculated via Pearson’s correlation between the time series at each vertex and the ROIs, and the top 10% of the correlations remained. Connectivity profiles were then averaged for all subjects, and von Mises-Fisher clustering methods and Hungarian matching were conducted to organize clusters of the cortex into 17 networks^82^. The parcellated maps were then spatially normalized to Montreal Neurological Institute (MNI) standardized space.

### Thalamic and cerebellar network parcellation and CTC integrative map generation

For thalamic and cerebellar network parcellation, the preprocessed data were projected to MNI standardized space, and these data were then resampled to a 2 × 2 × 2 mm^3^ voxel dimension. The thalamic and cerebellar network maps were generated separately to calculate the FC of the thalamus and of the cerebellum to cortical networks, respectively. Similar to cortical parcellation, individual parcellations of the thalamus and cerebellum for each subject were carried out first. We used the Harvard-Oxford atlas for the thalamus mask by combining the left and right thalamus both thresholded at 10% and used the FSL MNI FNIRT cerebellum atlas for the cerebellum mask. Pearson correlations were computed between each signal in each voxel of thalamic and cerebellar masks and the signal of each of the 17 networks from the group cortical map. The network assignment for each voxel was determined via a winner-take-all method, in which the network with the highest positive correlation value was assigned to the voxel^52,83^.

We then labeled each voxel to the cortical network that had been most frequently assigned across each voxel in the individual thalamic and cerebellar parcellation map. As demonstrated in Fig. 1, the final CTC map was created by integrating three different group parcellation maps: the cortical network group parcellation map, the thalamic group parcellation map, and the cerebellar group network map. The final CTC map was separated into 17 segregated network ROI maps.

### Stroop Color Word interference performance

The SCW Test was administered outside the scanner. The SCW test typically consists of three subtests: the color (C) trial, the word (W) trial, and the CW trial. During the C subtest, participants are presented with a series of colored squares on a sheet of paper and are asked to name the color of each square as quickly and accurately as possible. Similarly, during the W subtest, participants are given a sheet of paper with the names of colors written in black ink and are asked to read the words as quickly and accurately as possible.

The current analysis utilized the performance in the CW trial, which included the number of errors and number of self-corrections, as it reflects more complex cognitive functioning that encompasses broader domains, including executive functioning, working memory, attention, and conflict monitoring^84^. During the CW trials, the participants were given the stimuli card with the name of a color written in a different color ink; then, participants were asked to name the color of the ink aloud. The participants’ SCW-CW test performance is summarized in Table 1.

### Analysis of between-network connectivity and neurocognitive performance

Following our hypothesis, we focused on the higher-order cognitive networks, which resulted in a total of 11 network ROI (DAN (2), DMN (3), CEN (4), and SAL (2)) maps. To assess the group differences in the FC between the integrative CTC networks, an 11 × 11 connectivity matrix was generated to calculate the functional correlations between all pairs of the network ROIs. Using Fisher’s z transformation, the correlation coefficients were transformed into normally distributed values. We used a data-driven hierarchical clustering method based on functional similarity^85^ to generate clusters of ROI pairs for all 11 ROIs. The functional connectivity of all 55 connections between pairs of ROIs was then calculated using multivariate parametric GLM analysis^86^. With age controlled as a covariate, the cluster-level threshold and connection-level threshold were set at FDR-corrected *p* < 0.05.

To investigate the association between the between-network connectivity that showed significant group differences and neurocognitive functioning in patients, a partial correlation was performed controlling for IQ using IBM Statistics SPSS (Version 25). All statistical tests were performed two-sided.

## Data Availability

The data that support the results of this study are available from the corresponding author upon reasonable request. The data are not available because they contain information that could compromise the privacy of the research participants.

## Code availability

The code for data analysis is available from the corresponding author upon reasonable request.

## References

[1] Owen MJ, Sawa A, Mortensen PB (2016). Schizophrenia. Lancet.

[2] Hwang WJ (2019). Global and specific profiles of executive functioning in prodromal and early psychosis. Front. Psychiatry.

[3] Addington J, Brooks BL, Addington D (2003). Cognitive functioning in first episode psychosis: initial presentation. Schizophr. Res..

[4] Bora E, Murray RM (2014). Meta-analysis of cognitive deficits in ultra-high risk to psychosis and first-episode psychosis: do the cognitive deficits progress over, or after, the onset of psychosis?. Schizophr. Bull..

[5] Green MF, Horan WP, Lee J (2019). Nonsocial and social cognition in schizophrenia: current evidence and future directions. World Psychiatry.

[6] Harvey PD (2022). Cognitive dysfunction in schizophrenia: an expert group paper on the current state of the art. Schizophr. Res. Cogn..

[7] Raffard S, Bayard S (2012). Understanding the executive functioning heterogeneity in schizophrenia. Brain Cogn.

[8] McCutcheon RA, Keefe RSE, McGuire PK (2023). Cognitive impairment in schizophrenia: aetiology, pathophysiology, and treatment. Mol. Psychiatry.

[9] Kadakia A (2022). Healthcare resource utilization and quality of life by cognitive impairment in patients with schizophrenia. Schizophr. Res. Cogn..

[10] Andreasen NC (1996). Schizophrenia and cognitive dysmetria: a positron-emission tomography study of dysfunctional prefrontal-thalamic-cerebellar circuitry. Proc. Natl. Acad. Sci. USA..

[11] Lungu O (2013). The incidence and nature of cerebellar findings in schizophrenia: a quantitative review of fMRI literature. Schizophr. Bull.

[12] Parvizi J (2009). Corticocentric myopia: old bias in new cognitive sciences. Trends Cogn. Sci..

[13] Antonucci LA (2021). Flexible and specific contributions of thalamic subdivisions to human cognition. Neurosci. Biobehav. Rev..

[14] Pergola G, Selvaggi P, Trizio S, Bertolino A, Blasi G (2015). The role of the thalamus in schizophrenia from a neuroimaging perspective. Neurosci. Biobehav. Rev..

[15] Dehghani N, Wimmer RD (2019). A computational perspective of the role of the thalamus in cognition. Neural Comput.

[16] Watanabe Y, Funahashi S (2012). Thalamic mediodorsal nucleus and working memory. Neurosci. Biobehav. Rev..

[17] Stoodley CJ, Schmahmann JD (2009). Functional topography in the human cerebellum: a meta-analysis of neuroimaging studies. Neuroimage.

[18] Stoodley CJ, Schmahmann JD (2010). Evidence for topographic organization in the cerebellum of motor control versus cognitive and affective processing. Cortex.

[19] Van Overwalle F, Baetens K, Marien P, Vandekerckhove M (2014). Social cognition and the cerebellum: a meta-analysis of over 350 fMRI studies. Neuroimage.

[20] Parnaudeau S, Bolkan SS, Kellendonk C (2018). The mediodorsal thalamus: an essential partner of the prefrontal cortex for cognition. Biol. Psychiatry.

[21] Parnaudeau S (2013). Inhibition of mediodorsal thalamus disrupts thalamofrontal connectivity and cognition. Neuron.

[22] Bolkan SS (2017). Thalamic projections sustain prefrontal activity during working memory maintenance. Nat. Neurosci..

[23] Rikhye RV, Gilra A, Halassa MM (2018). Thalamic regulation of switching between cortical representations enables cognitive flexibility. Nat. Neurosci..

[24] Schmahmann JD, Sherman JC (1998). The cerebellar cognitive affective syndrome. Brain.

[25] McAfee SS, Liu Y, Sillitoe RV, Heck DH (2021). Cerebellar Coordination of Neuronal Communication in Cerebral Cortex. Front Syst Neurosci.

[26] Steiner L (2020). Functional topography of the thalamo-cortical system during development and its relation to cognition. Neuroimage.

[27] Bernard JA (2013). Disrupted cortico-cerebellar connectivity in older adults. Neuroimage.

[28] Kim SE, Jung S, Sung G, Bang M, Lee SH (2021). Impaired cerebro-cerebellar white matter connectivity and its associations with cognitive function in patients with schizophrenia. NPJ Schizophr.

[29] Mouchet-Mages S (2011). Correlations of cerebello-thalamo-prefrontal structure and neurological soft signs in patients with first-episode psychosis. Acta Psychiatr. Scand.

[30] Cho KI (2016). Altered thalamo-cortical white matter connectivity: probabilistic tractography study in clinical-high risk for psychosis and first-episode psychosis. Schizophr. Bull..

[31] Cho KIK (2019). Microstructural changes in higher-order nuclei of the thalamus in patients with first-episode psychosis. Biol. Psychiatry.

[32] Ferri J (2018). Resting-state thalamic dysconnectivity in schizophrenia and relationships with symptoms. Psychol. Med..

[33] Hwang WJ (2022). Thalamic connectivity system across psychiatric disorders: current status and clinical implications. Biol. Psychiatry Glob. Open Sci..

[34] Wang HL, Rau CL, Li YM, Chen YP, Yu R (2015). Disrupted thalamic resting-state functional networks in schizophrenia. Front. Behav. Neurosci..

[35] Woodward ND, Karbasforoushan H, Heckers S (2012). Thalamocortical dysconnectivity in schizophrenia. Am. J. Psychiatry.

[36] Chen P, Ye E, Jin X, Zhu Y, Wang L (2019). Association between thalamocortical functional connectivity abnormalities and cognitive deficits in schizophrenia. Sci. Rep..

[37] Anticevic A (2015). Association of Thalamic Dysconnectivity and Conversion to Psychosis in Youth and Young Adults at Elevated Clinical Risk. JAMA Psychiatry.

[38] Woodward ND, Heckers S (2016). Mapping thalamocortical functional connectivity in chronic and early stages of psychotic disorders. Biol. Psychiatry.

[39] Kwak YB (2021). Mapping thalamocortical functional connectivity with large-scale brain networks in patients with first-episode psychosis. Sci. Rep..

[40] Smith SM (2009). Correspondence of the brain’s functional architecture during activation and rest. Proc. Natl. Acad. Sci. USA..

[41] Reineberg AE, Gustavson DE, Benca C, Banich MT, Friedman NP (2018). The relationship between resting state network connectivity and individual differences in executive functions. Front. Psychol..

[42] Beaty RE, Seli P, Schacter DL (2019). Network neuroscience of creative cognition: mapping cognitive mechanisms and individual differences in the creative brain. Curr. Opin. Behav. Sci..

[43] Hausman HK (2020). The role of resting-state network functional connectivity in cognitive aging. Front. Aging Neurosci..

[44] Hearne LJ, Mattingley JB, Cocchi L (2016). Functional brain networks related to individual differences in human intelligence at rest. Sci. Rep..

[45] Satterthwaite TD, Baker JT (2015). How can studies of resting-state functional connectivity help us understand psychosis as a disorder of brain development?. Curr. Opin. Neurobiol..

[46] Bassett DS, Xia CH, Satterthwaite TD (2018). Understanding the emergence of neuropsychiatric disorders with network neuroscience. Biol. Psychiatry Cogn. Neurosci. Neuroimaging.

[47] Adhikari BM (2019). Functional network connectivity impairments and core cognitive deficits in schizophrenia. Hum. Brain Mapp..

[48] Anhoj S (2018). Alterations of intrinsic connectivity networks in antipsychotic-naive first-episode schizophrenia. Schizophr. Bull.

[49] Dong D, Wang Y, Chang X, Luo C, Yao D (2018). Dysfunction of large-scale brain networks in schizophrenia: a meta-analysis of resting-state functional connectivity. Schizophr. Bull.

[50] Yamashita M (2022). Cognitive functions relating to aberrant interactions between task-positive and task-negative networks: resting fMRI study of patients with schizophrenia. Appl. Neuropsychol. Adult.

[51] Kim A (2022). Triple-network dysconnectivity in patients with first-episode psychosis and individuals at clinical high risk for psychosis. Psychiatry Investig.

[52] Raut RV, Snyder AZ, Raichle ME (2020). Hierarchical dynamics as a macroscopic organizing principle of the human brain. Proc. Natl. Acad. Sci. USA..

[53] Hwang K, Bertolero MA, Liu WB, D’Esposito M (2017). The human thalamus is an integrative hub for functional brain networks. J. Neurosci..

[54] Buckner RL, Krienen FM, Castellanos A, Diaz JC, Yeo BT (2011). The organization of the human cerebellum estimated by intrinsic functional connectivity. J. Neurophysiol..

[55] Yuan R (2016). Functional topography of the thalamocortical system in human. Brain Struct. Funct..

[56] Kim M (2023). Large-scale thalamocortical triple network dysconnectivities in patients with first-episode psychosis and individuals at risk for psychosis. Schizophr. Bull..

[57] Shinn AK, Baker JT, Lewandowski KE, Ongur D, Cohen BM (2015). Aberrant cerebellar connectivity in motor and association networks in schizophrenia. Front. Hum. Neurosci..

[58] Yeo BT (2011). The organization of the human cerebral cortex estimated by intrinsic functional connectivity. J Neurophysiol.

[59] Iglesias JE (2018). A probabilistic atlas of the human thalamic nuclei combining ex vivo MRI and histology. Neuroimage.

[60] Diedrichsen J, Balsters JH, Flavell J, Cussans E, Ramnani N (2009). A probabilistic MR atlas of the human cerebellum. Neuroimage.

[61] Xue A (2021). The detailed organization of the human cerebellum estimated by intrinsic functional connectivity within the individual. J. Neurophysiol..

[62] O’Reilly JX, Beckmann CF, Tomassini V, Ramnani N, Johansen-Berg H (2010). Distinct and overlapping functional zones in the cerebellum defined by resting state functional connectivity. Cereb. Cortex.

[63] Habas C (2009). Distinct cerebellar contributions to intrinsic connectivity networks. J. Neurosci..

[64] Ponce GV, Klaus J, Schutter D (2021). A brief history of cerebellar neurostimulation. Cerebellum.

[65] Bolkan SS (2018). Publisher correction: thalamic projections sustain prefrontal activity during working memory maintenance. Nat. Neurosci..

[66] Fan Y (2015). Functional connectivity-based parcellation of the thalamus: an unsupervised clustering method and its validity investigation. Brain Connect.

[67] O’Neill A, Mechelli A, Bhattacharyya S (2019). Dysconnectivity of large-scale functional networks in early psychosis: a meta-analysis. Schizophr. Bull.

[68] Chan SY (2022). Dynamic and progressive changes in thalamic functional connectivity over the first five years of psychosis. Mol. Psychiatry.

[69] Wagner G (2013). Structural basis of the fronto-thalamic dysconnectivity in schizophrenia: a combined DCM-VBM study. Neuroimage Clin.

[70] Chen MH (2020). Cortico-thalamic dysconnection in early-stage schizophrenia: a functional connectivity magnetic resonance imaging study. Eur. Arch. Psychiatry Clin. Neurosci..

[71] Wu G (2022). Imbalance between prefronto-thalamic and sensorimotor-thalamic circuitries associated with working memory deficit in schizophrenia. Schizophr. Bull.

[72] Menon V (2011). Large-scale brain networks and psychopathology: a unifying triple network model. Trends Cogn. Sci..

[73] Menon B (2019). Towards a new model of understanding - the triple network, psychopathology and the structure of the mind. Med. Hypotheses.

[74] Supekar K, Cai W, Krishnadas R, Palaniyappan L, Menon V (2019). Dysregulated brain dynamics in a triple-network saliency model of schizophrenia and its relation to psychosis. Biol. Psychiatry.

[75] Gong Q (2017). Network-Level Dysconnectivity in Drug-Naive First-Episode Psychosis: Dissociating Transdiagnostic and Diagnosis-Specific Alterations. Neuropsychopharmacology.

[76] Wotruba D (2014). Aberrant coupling within and across the default mode, task-positive, and salience network in subjects at risk for psychosis. Schizophr. Bull.

[77] Fair DA (2009). Functional brain networks develop from a “local to distributed” organization. PLoS Comput. Biol..

[78] Kundu P (2018). The integration of functional brain activity from adolescence to adulthood. J. Neurosci..

[79] Power JD (2014). Methods to detect, characterize, and remove motion artifact in resting state fMRI. Neuroimage.

[80] Yum, T. et al. *The manual of Korean-Wechsler adult intelligence scale*. (1992).

[81] Doppelt JE (1956). Estimating the full scale score on the Wechsler adult intelligence scale from scores on four subtests. J Consult Psychol.

[82] Lashkari D, Vul E, Kanwisher N, Golland P (2010). Discovering structure in the space of fMRI selectivity profiles. Neuroimage.

[83] Ji JL (2019). Mapping the human brain’s cortical-subcortical functional network organization. Neuroimage.

[84] Jensen AR, Rohwer WD (1966). The Stroop color-word test: a review. Acta Psychol (Amst).

[85] Sørensen T (1948). A method of establishing groups of equal amplitude in plant sociology based on similarity of species and its application to analyses of the vegetation on Danish commons. Biologiske Skrifter / Kongelige Danske Videnskabernes Selskab.

[86] Jafri MJ, Pearlson GD, Stevens M, Calhoun VD (2008). A method for functional network connectivity among spatially independent resting-state components in schizophrenia. Neuroimage.

